# Acute Cold Exposure Cell-Autonomously Reduces mTORC1 Signaling and Protein Synthesis Independent of AMPK

**DOI:** 10.3390/cells15010065

**Published:** 2025-12-30

**Authors:** Benjamin Y. Sung, Eliza J. Ford, Daniel J. Foster, Kyler J. Fullmer, Cosette Cromwell, Benoit Viollet, David M. Thomson

**Affiliations:** 1 Department of Cell Biology & Physiology, Brigham Young University, Provo, UT 84602, USA; 2 Institut Cochin, Université Paris Cité, CNRS, Inserm, 75014 Paris, France; benoit.viollet@inserm.fr

**Keywords:** cryotherapy, AMPK, mTORC1, skeletal muscle, protein synthesis, injury recovery

## Abstract

Cryotherapy is a commonly used strategy for skeletal muscle recovery, although the efficacy of its use has been controversial. Therefore, more research is needed to understand under what circumstances it should be used. This study aimed to examine the cell-autonomous effects of acute cold exposure on primary mouse myoblasts, focusing on metabolic signaling through the AMPK/mTORC1 pathway. In it, we hypothesized that cold exposure (COLD) would impair myoblast proliferation, differentiation, and protein synthesis in an AMPK-dependent manner. Wild-type (WT) and AMPK double-knockout (dKO) myoblast cultures were treated at 37 °C or 26 °C to evaluate AMPK-dependent effects. As expected, 30 min of cold exposure activated AMPK and decreased mTORC1 activity and protein synthesis; however, mTORC1 and protein synthesis were downregulated independently of AMPK activation. Additionally, cold exposure suppressed proliferation 6 h post-treatment in WT, but not dKO, myoblasts. On the other hand, in differentiated WT and dKO cells, cold treatment did not influence myotube size, although dKO myotubes exhibited decreased fusion index and increased size compared to WT. These findings offer new insights into the cell-autonomous metabolic effects of cryotherapy in skeletal muscle and indicate that while COLD-induced AMPK activation contributes to impaired myoblast proliferation, AMPK is not necessary for the COLD-induced inhibition of the mTORC1 pathway and protein synthesis.

## 1. Introduction

Cryotherapy (e.g., cold baths or icing) has been used for years as a therapeutic modality to enhance recovery from injury and exercise-associated muscle and connective tissue damage. The rationale for cryotherapy is based on its well-established analgesic effect and its ability to reduce inflammation and metabolic rate, which are thought to limit secondary injury resulting from hypoxia [1,2]. However, its benefits have been questioned recently as our understanding of the crucial role inflammation plays in tissue regeneration and response to exercise has evolved [3,4,5]. This, combined with recent work showing that cold exposure reduces protein synthesis and stimulates protein catabolism, provides a counter-rationale for its use in muscle repair and recovery from injury or exercise [6]. Thus, the net effect of cold exposure on skeletal muscle metabolism and repair remains controversial.

At the systemic level, cold exposure significantly burdens homeothermic organisms by increasing energy demands to preserve core body temperature [7]. To adapt, skeletal muscle undergoes metabolic adjustments to favor catabolic processes such as lipolysis [8] and glycogenolysis [9], while suppressing protein synthesis [6,10], mitochondrial biogenesis [11], and satellite cell activation [6]. At least part of this metabolic response is due to hormonal and systemic factors (e.g., epinephrine/sympathetic nervous system activation). However, direct, cell-autonomous effects of cold are likely involved as well, although these effects are not currently well defined.

The mechanistic target of rapamycin complex 1 (mTORC1) is a central regulator of skeletal muscle growth and regeneration, promoting protein synthesis and cellular growth while inhibiting autophagy under energy- or nutrient-rich conditions [12]. Evidence from long-term cold exposure experiments indicates that mTORC1 signaling is reduced under cold stress, as shown by decreased phosphorylation of mTORC1 targets involved in protein synthesis [13,14,15]. These findings suggest prolonged cold exposure can inhibit anabolic signaling and potentially interfere with muscle repair and regeneration. It is not clear, however, whether shorter-term therapeutically relevant cooling negatively affects mTORC1 signaling.

A likely upstream regulator of this effect is AMP-activated protein kinase (AMPK). This cellular energy sensor is activated in skeletal muscle during metabolic stress, such as exercise or nutrient deprivation. Upon activation, AMPK shifts cellular activity from energy-consuming anabolic processes toward energy-generating catabolic pathways [16,17], partly by inhibiting mTORC1 [18,19,20,21]. Skeletal muscle AMPK activity increases in response to cold stress [10,22], supporting the idea that AMPK may mediate the suppression of mTORC1 under these conditions. This mechanism may help explain how acute cold exposure leads to reduced protein synthesis and impaired muscle regeneration. On the other hand, while acute AMPK activation clearly suppresses mTORC1 and protein synthesis, recent evidence suggests that anabolic signaling may be enhanced after skeletal muscle cells recover from AMPK activation [23]. Therefore, cold-induced AMPK activation and mTORC1 inhibition may ultimately lead to increased anabolism and mTORC1 signaling following recovery from AMPK activation.

In this paper, we test the hypothesis that (1) acute exposure of skeletal muscle myoblasts to cold temperatures acutely inhibits mTORC1 activity and protein synthesis in a cell-autonomous and AMPK-dependent manner, and (2) myoblast proliferation and differentiation to myotubes are subsequently enhanced in an AMPK-dependent manner after recovery from acute cold exposure. Our findings provide valuable insight into the direct effects of cryotherapy on skeletal muscle cell metabolism and repair.

## 2. Materials and Methods

### 2.1. Cell Culture

Immortalized myoblasts were obtained from the gastrocnemius and tibialis anterior muscles of 4-week old wild-type (WT) and AMPK α1-α2 double-knockout (AMPK dKO) mice in compliance with the guidelines established by the European Convention for the Protection of Laboratory Animals and approved by the Committee of the Ethics of Animal Experiments Paris Descartes *n*°34 [24]. The cells were proliferated at 37 °C and 5% CO_2_ in growth media [GM; Dulbecco’s Modified Eagle Medium (DMEM)-F12 (Cytiva-SH30023.01; Marlborough, MA, USA), 10% Fetal Bovine Serum, (Thermo-Fisher-FB12999102; Waltham, MA, USA), 1% penicillin–streptomycin–amphotericin (PSA, Cytiva-SV30079.01; Marlborough, MA, USA), 0.5% GlutaMAX (Thermo Fisher-35050-061, Waltham, MA, USA), and 5 ng/mL fibroblast growth factor 2] on plates precoated with 2% gelatin. Where applicable, myotubes were differentiated by switching near-confluent (~85–95% by subjective assessment) myoblasts to differentiation media consisting of 5% horse serum and 1% PSA in DMEM. As indicated in the individual experiments, the cells were incubated continuously for the time periods indicated at 37 °C or treated at 26 °C, which is approximately the intramuscular temperature achieved at a depth of 3″ under the skin after 30 min of cryotherapy (icing + compression) in human quadricep muscles [25].

### 2.2. Measurement of Protein Synthesis and Cell Signaling

WT and dKO myoblasts were seeded in 6-well plates and allowed to proliferate to ~85–95% confluence. Some plates were then incubated at 26 °C and 5% CO_2_ for 1 h, while the other cells remained at 37 °C continuously before lysis and cell collection. At 30 min prior to lysis, 1 μM puromycin was added to the culture media to assess protein synthesis using the SUnSET method [26]. In a separate experiment, WT and dKO myoblasts were lysed at 6 or 24 h after exposure to 26 °C.

Cells were lysed in HEPES lysis buffer with protease and phosphatase inhibitors (50 mM HEPES, 150 mM NaCl, 1.5 mM MgCl_2_, 1 mM EGTA, 15 mM sodium pyrophosphate, 100 mM β-glycerophosphate, 25 mM NaF, 1 mM sodium orthovanadate, 1% Triton X-100, 1 mM benzamidine, 5 μg/mL soybean trypsin inhibitor, 150 μM PMSF, 1 μM Pepstatin-A). Lysates were clarified by centrifugation at 10,000× *g* for 10 min at 4 °C, and supernatants were used for subsequent analysis by Western blotting. Puromycin incorporation into nascent protein, and markers of AMPK and mTORC1 signaling were then detected by Western blotting, as described below.

In a separate experiment, WT and dKO myoblasts were seeded in 6-well plates, exposed to a temperature of 26 °C for 1 h, then lysed 6 or 24 h later.

### 2.3. Cell Proliferation Assay

Cell proliferation was determined by measuring the incorporation of bromodeoxyuridine (BrDU) into newly synthesized DNA. Three hundred myoblasts were seeded per well in 96-well plates. Then, 27 h later, 10 μM BrDU was added to the media in each well, and the cells were incubated for an additional 2 h, after which they were fixed and stained for total (DAPI) and proliferating (BrDU) nuclei (see staining protocol below). Cells in the cooling groups were transferred to a 26 °C incubator for 1 h, starting at 25, 7, or 1 h before fixation and staining (e.g., the cells were fixed 24, 6, or 0 h after cold exposure).

The cells were fixed with cold 70% ethanol for 5 min, permeabilized with 0.3% Triton X-100 in PBS for 10 min, DNA-denatured with 2N HCl for 20 min, and blocked for 1 h in 5% normal goat serum. The cells were then probed overnight with a 1:100 dilution of mouse anti-BrDU antibody [Developmental Studies Hybridoma Bank (DSHB) #G3G4], then probed for 1 h in a 1:100 dilution of secondary antibody (AlexaFluor 488 rabbit anti-mouse IgG_1_, Southern Biotech #6170-30 (Birmingham, AL, USA)), and finally with 1 μg/mL DAPI for 5 min. The BrDU-positive and -negative nuclei were imaged using a fluorescence microscope. A total of >170 nuclei were counted for each of the three independent replicates using ImageJ software (v.154r).

### 2.4. Myotube Differentiation Assay

A total of 5000 myoblasts per well were seeded on 12-well plates and allowed to proliferate for 2 days to ~85–95% confluence by subjective assessment; they were then differentiated for 4 days. Cells were either incubated continuously at 37 °C or exposed for 1 h per day to a temperature of 26 °C throughout the proliferation and differentiation period. Then, 24 h after the final exposure to reduced temperature, the cells were fixed for 10 min in 4% paraformaldehyde, permeabilized with 0.3% Triton X-100 in PBS for 10 min, and incubated overnight at 4 °C in a 1:50 dilution of anti-myosin heavy-chain primary antibody (DSHB #MF20). The cells were then incubated for 1 h at room temperature in the dark with a 1:100 dilution of AlexaFluor488-labeled secondary antibody and incubated with 1 μg/mL DAPI for 5 min at room temperature in the dark. For fusion index measures, seven fields were imaged for each of the three replicates per group; then, the nuclei associated or not with MHC-positive cells (1200–2300 nuclei per sample) were counted using ImageJ, v1.54r. Fusion index was calculated as the number of nuclei in MHC-positive myotubes/total nuclei per field. Myotube diameter was calculated as the average of measurements at 5 locations per myotube from 4 fields for each of the 3 replicates per group (120 myotubes measured per replicate).

### 2.5. Western Blotting

The protein concentration of the clarified cell lysates was determined using the DC Protein Assay (Bio-Rad, Hercules, CA, USA). Equal amounts of protein were separated by electrophoresis and transferred to polyvinylidene fluoride (PVDF) membranes. Proper transfer and equal protein loading were verified by Ponceau-S staining of the PVDF membranes. Primary antibodies [Puromycin (Millipore MABE343, Bedford, MA, USA), phospho-ACC (Cell Signaling #3661, Danvers, MA, USA), ACC (Cell Signaling #3676), phospho-S6K (Cell Signaling #9234), S6K (Cell Signaling #2708), phospho-S6 (Cell Signaling #4858), S6 (Cell Signaling #2217)] were applied for either 4 h at room temperature or overnight at 4 °C. Membranes were then incubated in near-infrared fluorescent secondary antibodies [anti-mouse (Jackson Immunoresearch #115-625-174, West Grove, PA, USA) or anti-rabbit (ThermoFisher #SA535571, Waltham, MA, USA)] for 1 h at room temperature. Labeled proteins were visualized using a near-infrared scanner (Licor Odyssey DLx), and resulting band intensities were quantified using ImageStudio 5.2 software (LICORbio, Lincoln, NE, USA)

### 2.6. Statistics

Statistical analysis and generation were performed using BioRender (biorender.com). Data are presented as means ± SEM. Unpaired *t*-tests were used to assess differences between WT and AMPK dKO untreated control (CTRL) cells. For comparisons with two factors (genotype and temperature), two-way ANOVA was performed, with the Bonferroni post hoc test applied when an interaction was detected. Comparisons of only one factor were assessed using one-way ANOVA with Dunnett multiple-comparisons post hoc testing. For ANOVA analysis, normality within groups was confirmed using the Shapiro–Wilk test, and equal variance with the Levene test. Significance level was set at 0.05.

## 3. Results

### 3.1. Acute Cold Exposure Limits Myoblast Protein Synthesis and mTORC1 Signaling in an AMPK-Independent Manner

**Figure 1:**
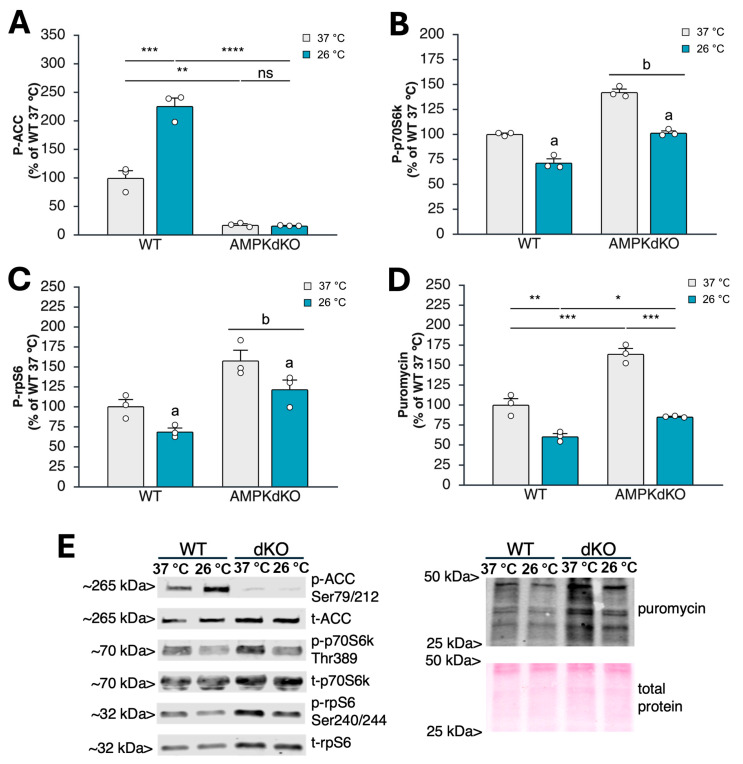
Acute cold exposure downregulates protein synthesis in an AMPK-independent manner. Wild-type (WT) and AMPK double-knockout (dKO) myoblasts [*n* = 3 biological replicates (from separate cultures)/group] were incubated at 37 °C or 26 °C for 1 h, with the addition of puromycin for the final 30 min of incubation. The cells were then harvested and assayed for indicators of AMPK activity, anabolic signaling through mTORC1, and protein synthesis by Western blotting. (**A**) Levels of phosphorylated ACC (Ser79/212; p-ACC); (**B**) levels of phosphorylated p70S6k (Thr389; p-p70S6k); (**C**) levels of phosphorylated S6 (Ser240/244; p-rpS6); (**D**) levels of puromycin incorporation (indicative of protein synthesis); (**E**) representative blots for phosphorylated (p-ACC, p-p70S6k, p-rps6) and total (t-ACC, t-p70S6k, t-rpS6, puromycin) proteins shown in (**A**–**D**), and representative Ponceau stain for total protein showing equal protein loading and effective transfer. Data are means +/− S.E.M. Significant differences identified by 2 × 2 factorial ANOVA are indicated on the graph. Where only main effects without an interaction were detected, a = main effect of temperature; b = main effect of genotype. Where a significant interaction was detected, Bonferroni post hoc analysis was performed to identify significant differences between individual groups: * *p* ≤ 0.05; ** *p* ≤ 0.01; *** *p* ≤ 0.001; **** *p* ≤ 0.0001; ns = no significant difference.

To determine if a therapeutically relevant duration of cooling suppresses anabolic signaling and protein synthesis in an AMPK-dependent manner, we incubated WT and dKO myoblasts at 37 °C or 26 °C for 1 h. As expected, ACC phosphorylation at Ser79/212 was almost completely absent in dKO cells (Figure 1A,E). This is consistent with the widespread use of this phosphorylation site as a reliable readout for in vivo AMPK activity [27]. Consistent with previous work [24], anabolic signaling downstream of mTORC1 was significantly elevated in dKO myoblasts, as shown by increased p70S6K (Figure 1B,E) and rpS6 (Figure 1C,E) phosphorylation. Protein synthesis was likewise increased in dKO cells (Figure 1D,E), consistent with the enhanced anabolic signaling via mTORC1 that we observed.

In WT myoblasts, cooling the cells to 26 °C increased ACC phosphorylation by 126.1 ± 13.9% (Figure 1A). As expected, ACC phosphorylation did not respond to cold exposure in cells lacking AMPK (Figure 1A). Levels of phospho-p70S6k (Figure 1B) and phospho-rpS6 (Figure 1C) decreased in WT myoblasts at 26 °C by 28.5 ± 4.0% and 31.0 ± 4.5%, respectively. This was consistent with a temperature-dependent decline in protein synthesis of 29.5 ± 3.4% at 26 °C (Figure 1D). Similar or greater reductions in anabolic signaling and protein synthesis were observed in dKO myoblasts (Figure 1C–E), indicating that the decrease in mTORC1 signaling and protein synthesis with cold exposure is not dependent on AMPK, at least in isolated cell culture. Similar findings were observed in differentiated myotubes, where p70S6k phosphorylation was suppressed with exposure to 26 °C in both WT and dKO myotubes, despite the lack of AMPK activation in both genotypes with cooling (Figure S1).

### 3.2. Acute Exposure to Cold Drives an AMPK-Dependent Decrease in Myoblast Proliferation Rate

**Figure 2:**
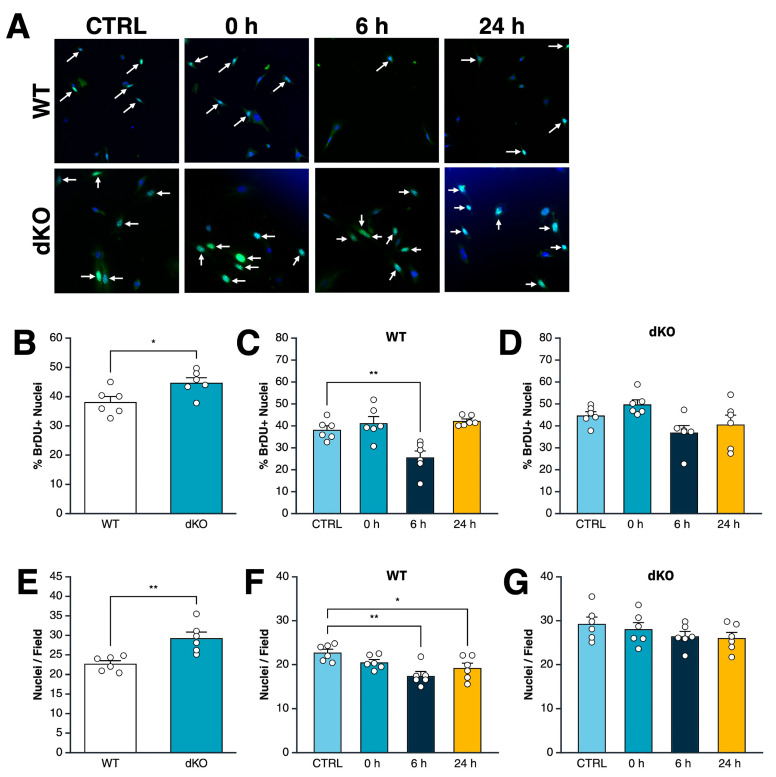
Cold exposure causes an AMPK-dependent decrease in myoblast proliferation rate. Myoblasts were proliferated for 29 h after seeding. Control (CTRL)-treated myoblasts were incubated continuously throughout the proliferation period at 37 °C, while the other cells were incubated at 26 °C for 1 h ending at 0, 6, or 24 h before staining. The 6 and 24 h groups were returned to 37 °C for the remaining time until fixation and staining. Bromodeoxyuridine (BrDU) was added for the final 2 h of incubation, after which the cells were fixed and nuclei stained with DAPI, and proliferating nuclei with BrDU. (**A**) Representative BrDU staining of wild-type (WT) and AMPK double-knockout (dKO) myoblasts at 0, 6, or 24 h after 1 h of cold exposure at 26 °C, or at 37 °C control. DAPI (total nuclei) is shown in blue and BrDU (proliferating nuclei) in green. (**B**) Percent BrDU-positive nuclei in WT vs. dKO CTRL-treated myoblasts. (**C**,**D**) Percent BrDU-positive nuclei at indicated timepoints for WT and dKO myoblasts, respectively. (**E**) Total nuclei per field for WT and dKO myoblasts. (**F**,**G**) Total nuclei per field at the indicated timepoints for both WT and dKO myoblasts. Significant differences between indicated groups by *t*-test (**B**,**E**) or ANOVA (**C**,**D**,**F**,**G**) denoted by * = *p* < 0.05; ** = *p* < 0.01. Data are means +/− S.E.M. *n* = 3 biological replicates (from separate cultures)/group.

To determine whether cold exposure impacts myoblast proliferation and if AMPK mediates this effect, we compared WT and dKO myoblast proliferation immediately, 6 h, or 24 h after a 1 h bout of cooling to 26 °C against proliferation in cells maintained continuously at 37 °C. Under control (37 °C) treatment, dKO cells exhibited significantly greater proliferation than WT cells (Figure 2B). Following cold exposure, WT cells showed a significant reduction in proliferation rate at 6 h relative to WT controls. Proliferation was restored to control treatment levels at 24 h, indicating the effect is transient (Figure 2C). In contrast, dKO cells showed no significant changes in proliferation at any timepoint post-cold exposure, indicating that AMPK is required for the transient suppression of proliferation observed in WT cells (Figure 2D).

We measured phosphorylation of ACC (reflective of AMPK activity) and rpS6 (reflective of anabolic signaling downstream of mTORC1) at 6 and 24 h after cooling, but observed no difference in WT or dKO myoblasts compared to corresponding CTRL-treated samples (Figure S2).

Consistent with the findings from the BrDU assay, CTRL-treated dKO cells displayed significantly higher nuclei counts than WT, aligning with their increased proliferative ability (Figure 2E). In WT cells, nuclei counts were significantly lower at both 6 and 24 h after cold exposure (Figure 2F), consistent with a period of suppressed proliferation observed in the BrDU assay. In contrast, dKO cell counts were not affected by cold exposure (Figure 2G). These findings indicate that cold exposure decreases proliferation in a manner dependent on AMPK, with the strongest effects between 6 and 24 h after treatment. dKO cells are resistant to these changes, supporting a role for AMPK in controlling myoblast division after cold stress.

### 3.3. Myoblast Differentiation Is Suppressed by the Lack of AMPK, but Is Unaffected by Intermittent Moderate Cold Exposure

**Figure 3:**
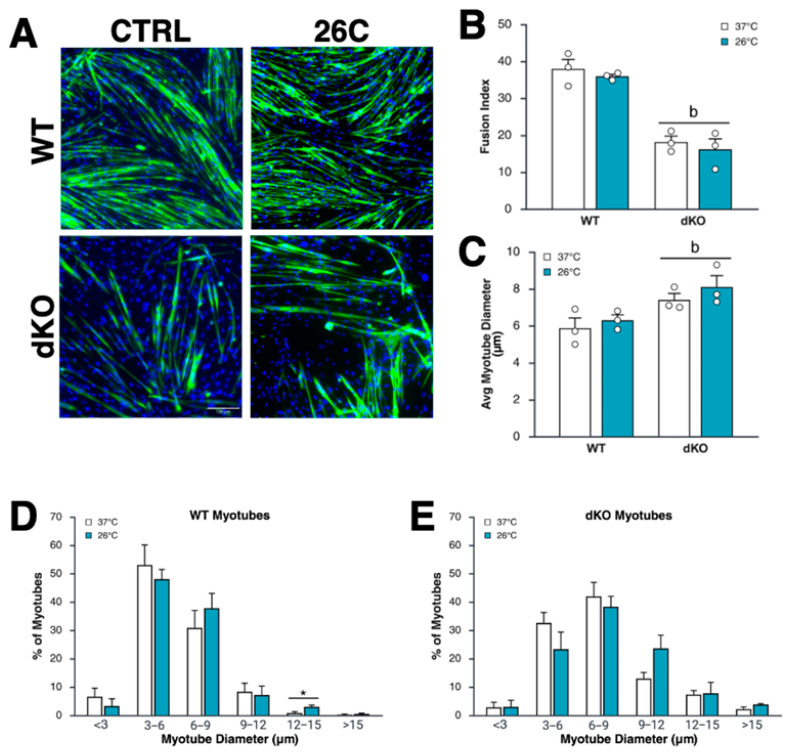
The lack of AMPK, but not intermittent cold exposure, suppresses myoblast differentiation. (**A**) Representative immunofluorescence staining of wild-type (WT) and AMPK double-knockout (dKO) primary myoblasts treated with daily 1 h cooling bouts at 26 °C (26C) vs. 37 °C throughout proliferation and differentiation (6 days total), then fixed and stained for DAPI (blue) and myosin heavy chain (green). (**B**) Fusion index (% MHC+ nuclei/total nuclei) of WT vs. dKO myoblasts. (**C**) Myotube size in μm. (**D**,**E**) Myotube diameter distribution of WT (**D**) and dKO (**E**) myotubes. Data are means +/− S.E.M. *n* = 3 biological replicates (from separate cultures)/group. “b” = Significant (*p* ≤ 0.01) main effect of genotype as determined by factorial ANOVA; * = significant difference (*p* < 0.05) between 37 °C and 26 °C as determined by *t*-test.

To evaluate the role of AMPK in the effect of short-term cooling on myotube differentiation and growth, we exposed myoblasts to daily one-hour cooling sessions at 26 °C versus 37 °C during proliferation (4 days) and differentiation (3 days). Differentiation decreased by 55–58% in dKO compared to WT myoblasts but was unaffected by cold exposure in either genotype (Figure 3A,B). Interestingly, although myoblast fusion was reduced in dKO myoblasts and there were noticeably fewer dKO myotubes per field, the average diameter of dKO myotubes increased by 26.9% relative to WT myotubes (7.4 ± 0.34 vs. 5.8 ± 0.55 μM, respectively; Figure 3A,C). This indicates that fewer but larger myotubes formed in dKO cultures. Myotube mean diameter remained unchanged by cooling in both genotypes (Figure 3A,C), although WT (but not dKO) myotubes treated at 26 °C showed a slight increase in the percentage of larger (12–15 µm) myotubes compared to control-treated myotubes (Figure 3D,E).

## 4. Discussion

Cryotherapy after exercise or injury is commonly used to aid in skeletal muscle recovery. However, recent research questions this practice, suggesting it may hinder hypertrophic and strength adaptations [6]. This could partly result from the cooling’s effect on protein synthesis. For example, 20 min of cold water immersion reduced post-resistance exercise protein synthesis in human muscle both immediately after a single workout and throughout two weeks of resistance training [28]. The mTORC1 signaling pathway is a primary stimulus for protein synthesis, and multiple studies have shown that cold exposure suppresses mTORC1 signaling in human muscle tissue [5,15,29].

AMPK is a well-known inhibitor of the mTORC1 pathway, preventing its stimulatory influence on protein synthesis [17]. After 1 h of cold exposure, ACC phosphorylation increased, indicating acute AMPK activation. At the same time, phosphorylation of both p70S6k and rpS6 decreased, showing a reduction in mTORC1 activity. Protein synthesis was also suppressed. However, when the same conditions were applied to AMPK double-knockout myoblasts, mTORC1 signaling remained suppressed, suggesting that cold exposure decreases mTORC1 signaling and protein synthesis through a mechanism that does not depend on AMPK. One possible mechanism for AMPK-independent mTORC1 inhibition is through activation of the temperature-sensitive transient receptor potential (TRP) channels. Activation of TRP subfamily M member 8 (TRPM8), a cold-induced channel, inhibits mTORC1 activity in keratinocytes similarly to the well-known mTORC1 inhibitor rapamycin [30]. Since TRPM8 is also expressed in skeletal muscle cells [31], it is a strong candidate for mediating cold-induced regulation of the mTORC1 pathway.

We found that 1 h of cold exposure caused a temporary decrease in WT myoblast proliferation 6 h afterward, with the proliferation rate returning to baseline levels after 24 h. Interestingly, the proliferation rate was not significantly affected immediately after the 1 h cooling period. Accordingly, total nuclei counts were similarly decreased for the 6 h and 24 h but not 0 h groups. The proliferation of dKO myoblasts was slightly higher compared to WT myoblasts, which has also been observed in mouse embryonic fibroblasts [32]. This aligns with the common effect of AMPK activation impairing cell proliferation, including in myoblasts [33]. Conversely, AMPK depletion can also hinder myoblast proliferation in culture [34] and satellite cell proliferation in vivo [35]; thus, AMPK’s impact on proliferation depends on the experimental context. Regardless, in contrast to WT cells, cold exposure did not affect proliferation in dKO myoblasts. These results suggest that while AMPK is not necessary for cold-induced suppression of protein synthesis, it is essential for the temporary inhibition of proliferation following cold exposure.

Fusion, the process by which myoblasts form multinucleated myotubes, is a key indicator of regenerative capacity. Prolonged (3–7 days) preconditioning of myoblasts with cold exposure may [36] or may not [36] enhance subsequent differentiation or fusion, but cold exposure throughout differentiation itself was reported not to affect myotube formation [37]. In this study, we exposed cells to cold for 1 h daily throughout proliferation and differentiation and observed no effect of temperature on the fusion or size of myotubes. Both AMPKα1 [38] and AMPK α2 [38] are known to support fusion and myonuclear accretion, and our results agree with this, as myotube fusion was significantly reduced in dKO cells, regardless of temperature. Interestingly, though there were fewer myotubes, individual myotube size increased in dKO cells, which aligns with the known role of AMPKα1 in suppressing muscle fiber size, likely through mTORC1 inhibition [24].

Taken together, our findings demonstrate that while cold-induced suppression of protein synthesis and mTORC1 signaling occurs independently of AMPK, the reduction in myoblast proliferation is AMPK-dependent. Additionally, myoblast differentiation and fusion are not enhanced following recovery from acute cold exposure. These findings clarify the cell-intrinsic role of AMPK in mediating the effect of cold in skeletal muscle.

## Data Availability

The original contributions presented in this study are included in the article/Supplementary Material. Further inquiries can be directed to the corresponding author.

## Supplementary Materials

The following supporting information can be downloaded at: https://www.mdpi.com/article/10.3390/cells15010065/s1, Figure S1: Acute cold exposure downregulates protein synthesis in an AMPK independent manner in primary mouse myotubes; Figure S2: ACC, p70S6k, and rpS6 phosphorylation is not different in myoblasts at 24 or 6 hours after cold exposure vs. uncooled cells; Figure S3: Complete, uncropped western blotting images.

## Abbreviations

The following abbreviations are used in this manuscript:

ACC	acetyl-CoA carboxylase
AMPK	AMP-activated protein kinase
dKO	AMPK alpha double knockout
mTORC1	mechansistic target of rapamycin, complex 1
p70S6k	ribosomal protein S6 kinase, of 70 kDa
rpS6	ribosomal protein S6
TRP	transient receptor potential
TRPM8	TRP subfamily M, member 8
WT	wild-type
