# Supplementary material for: Acute Cold Exposure Cell-Autonomously Reduces mTORC1 Signaling and Protein Synthesis Independent of AMPK
Source: Cells. 2025 Dec 30;15(1):65. doi: 10.3390/cells15010065 (PMC12785600; doi:10.3390/cells15010065)
Supplement: Supplementary file 1 [file cells-15-00065-s001.zip › SuppFig1.pdf]

Fig S1

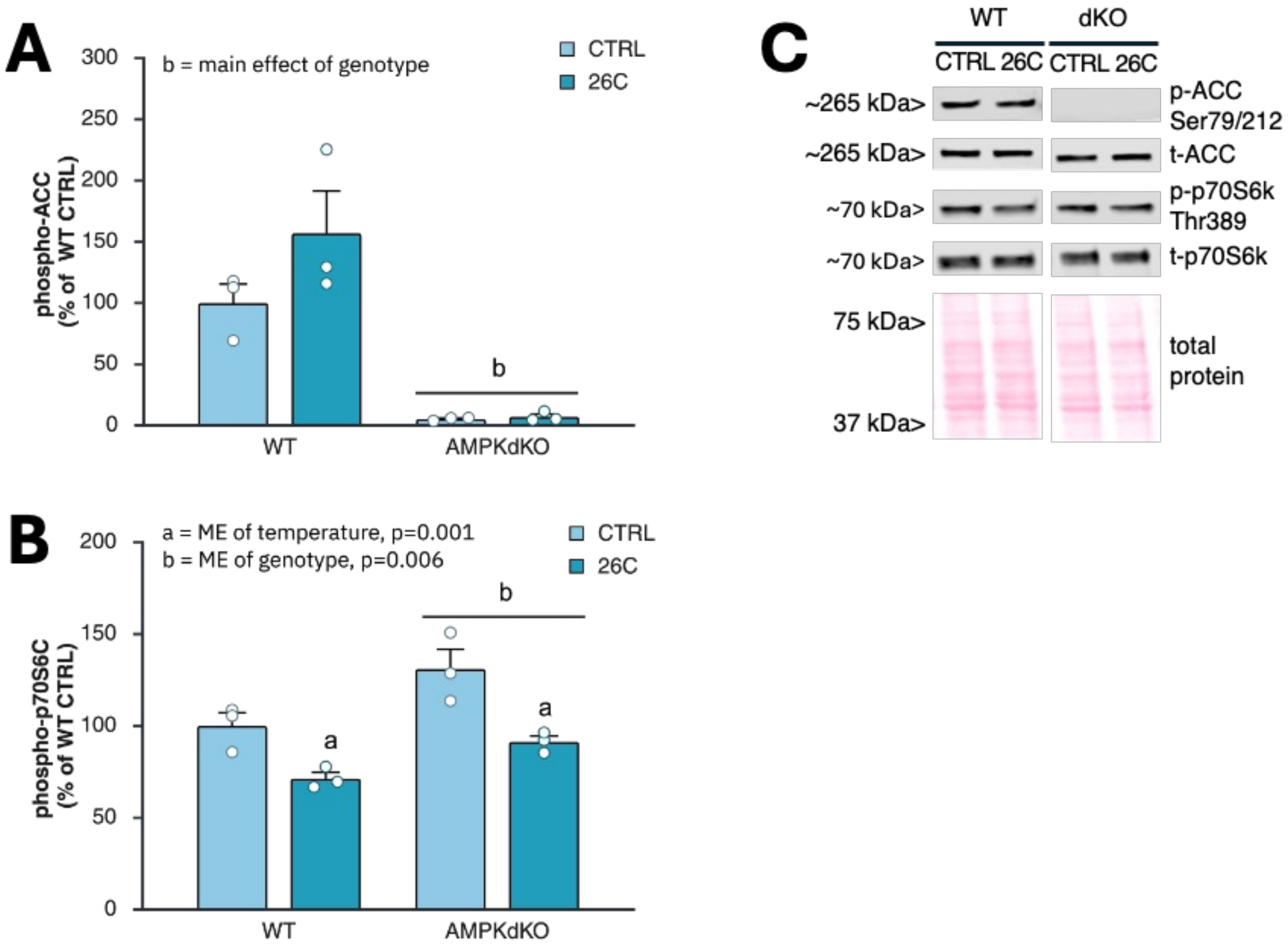

Figure S1. Acute cold exposure downregulates protein synthesis in an AMPK-independent manner in primary mouse myotubes. Wild type (WT) and AMPK double knockout (dKO) myotubes (n=3 independent samples/group) were incubated at 37°C (CTRL) or 26°C for 1 hour and assayed for ACC phosphorylation as an indicator of AMPK activity and p70S6k phosphorylation as an indicator of anabolic signaling through mTORC1by western blotting. (A) Levels of phosphorylated ACC (Ser79/212; p-ACC; (B) Levels of phosphorylated p70S6k (Thr389; p-p70S6k); (C) Representative blots for the data shown in A-B, and representative Ponceau stain for total protein showing equal protein loading and effective transfer. Data are means +/- S.E.M. Significant differences identified by 2 x 2 factorial ANOVA are indicated on the graph.
