# Supplementary material for: Acute Cold Exposure Cell-Autonomously Reduces mTORC1 Signaling and Protein Synthesis Independent of AMPK
Source: Cells. 2025 Dec 30;15(1):65. doi: 10.3390/cells15010065 (PMC12785600; doi:10.3390/cells15010065)
Supplement: Supplementary file 1 [file cells-15-00065-s001.zip › SuppFig2.pdf]

Fig S2

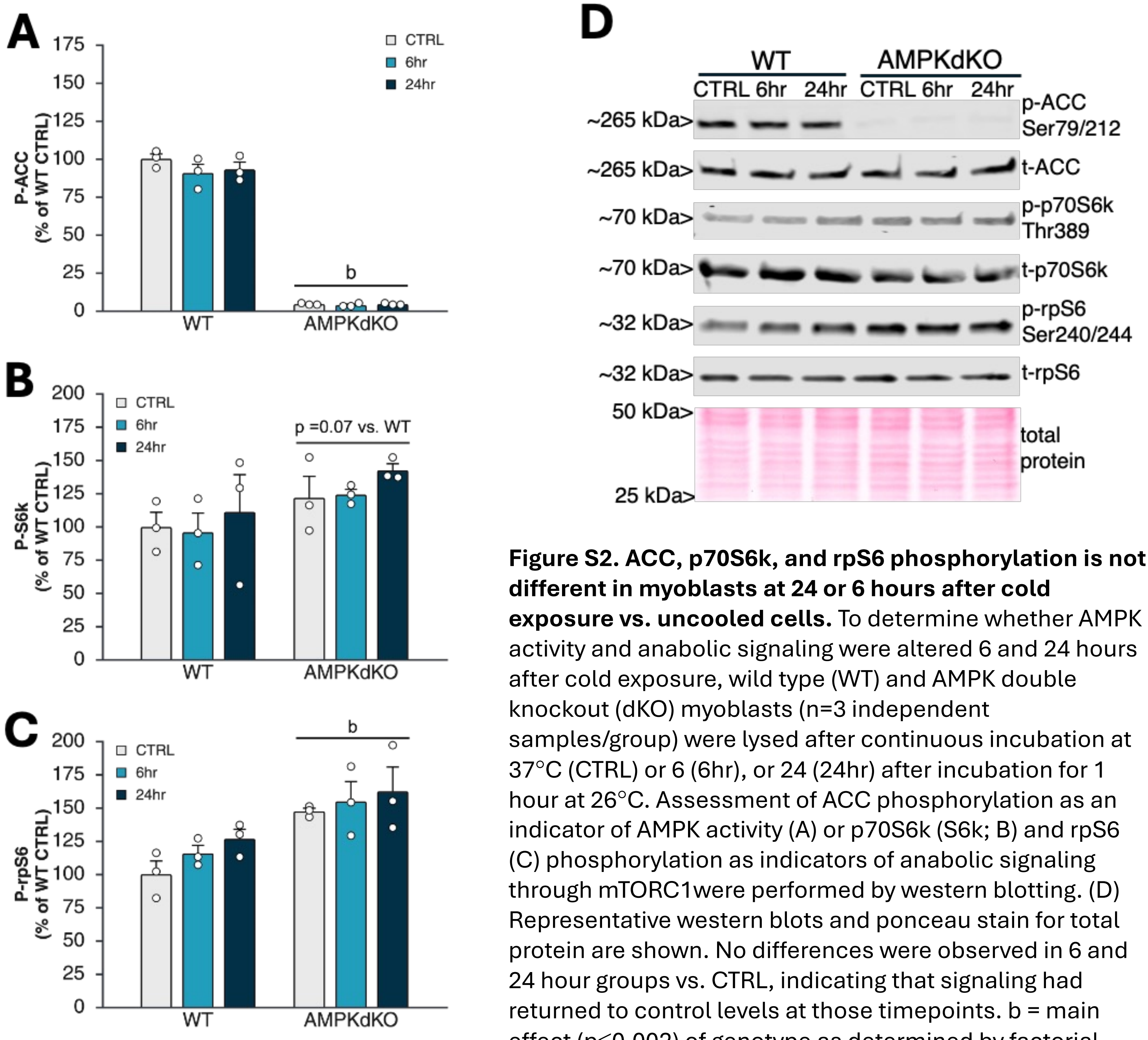

Figure S2. ACC, p70S6k, and rpS6 phosphorylation is not different in myoblasts at 24 or 6 hours after cold exposure vs. uncooled cells. To determine whether AMPK activity and anabolic signaling were altered 6 and 24 hours after cold exposure, wild type (WT) and AMPK double knockout (dKO) myoblasts (n=3 independent samples/group) were lysed after continuous incubation at 37°C (CTRL) or 6 (6hr), or 24 (24hr) after incubation for 1 hour at 26°C. Assessment of ACC phosphorylation as an indicator of AMPK activity (A) or p70S6k (S6k; B) and rpS6 (C) phosphorylation as indicators of anabolic signaling through mTORC1were performed by western blotting. (D) Representative western blots and ponceau stain for total protein are shown. No differences were observed in 6 and 24 hour groups vs. CTRL, indicating that signaling had returned to control levels at those timepoints. b = main effect ($p \leq 0.002$) of genotype as determined by factorial ANOVA analysis.
