# Supplementary material for: Acute Cold Exposure Cell-Autonomously Reduces mTORC1 Signaling and Protein Synthesis Independent of AMPK
Source: Cells. 2025 Dec 30;15(1):65. doi: 10.3390/cells15010065 (PMC12785600; doi:10.3390/cells15010065)
Supplement: Supplementary file 1 [file cells-15-00065-s001.zip › SuppFigS3.pdf]

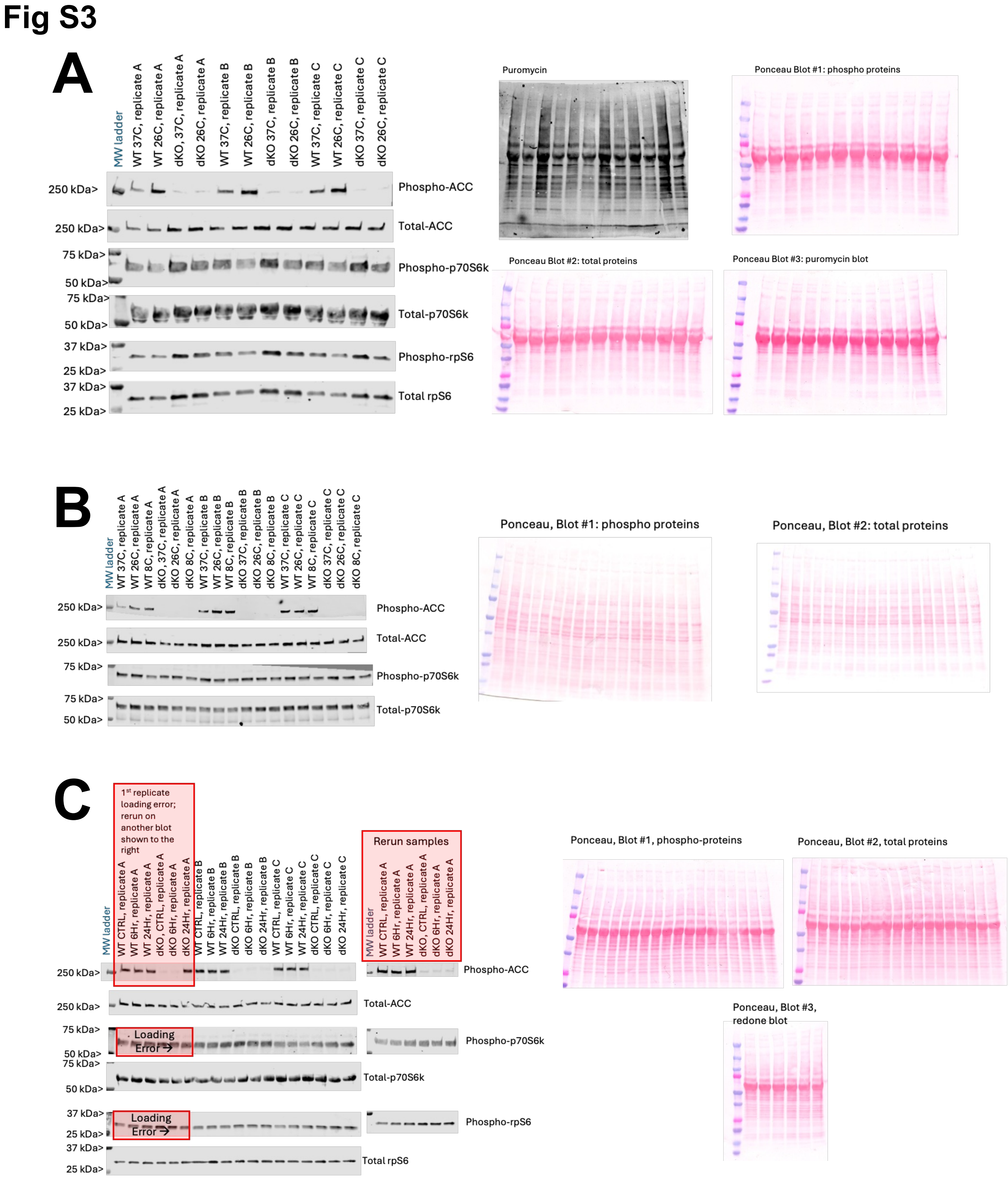

Figure S3. Complete, uncropped western blotting images. (A) Blots for the data in Figure 1. (B) Blots for the data in Figure S1. Note that an 8C group was included in the full blots, but was not within the scope of the manuscript. (C) Blots for the data in Figure S2. Note that the samples for replicate A were misloaded in the original gel (see the ACC+ band in one of the dKO lanes). The samples were reloaded correctly in an additional blot, labeled Rerun samples.
